# Follicular Helper T Cells in Systemic Lupus Erythematosus

**DOI:** 10.3389/fimmu.2018.01793

**Published:** 2018-08-03

**Authors:** Sun Jung Kim, Kyungwoo Lee, Betty Diamond

**Affiliations:** The Feinstein Institute for Medical Research, Northwell Health, New York, NY, United States

**Keywords:** follicular helper T cells, autoimmunity, transcription factors, human immunology, lupus models

## Abstract

CD4^+^ follicular helper T (Tfh) cells constitute a subset of effector T cells that participate in the generation of high-affinity humoral responses. They express the chemokine receptor CXCR5 and produce the cytokine IL-21, both of which are required for their contribution to germinal center formation. Uncontrolled expansion of Tfh cells is observed in various mouse models of systemic autoimmune diseases and in patients with these diseases. In particular, the frequency of circulating Tfh is correlated with disease activity and anti-DNA antibody titer in patients with systemic lupus erythematosus. Recent studies reveal functional diversity within the Tfh population in both humans and mice. We will summarize here the molecular mechanisms for Tfh cell generation, survival and function in both humans and mice, and the relationship between Tfh cells and autoimmune disease in animal models and in patients.

## Introduction

It has long been known that T cells are required for successful humoral immune responses (1). Upon stimulation, CD4^+^ naïve T cells differentiate into T helper (Th) 1, Th2, Th17, follicular helper T (Tfh), and regulatory T (Treg) cells. Each subset requires distinct activation signals and cytokine milieu during activation; each expresses a unique transcriptional profile with a unique master regulator and distinct effector cytokines; and each subset serves a different function in the immune response. Activation of naïve CD4^+^ T cells by antigen-presenting cells (APCs) together with IL-6 and IL-21 induces Tfh cells. Development of Tfh cells is required for an optimal antibody response, mainly through activation and maintenance of the germinal center (GC) response. Tfh cells express the master transcription factor, B cell lymphoma 6 (BCL6). This transcription factor distinguishes the Tfh compartment from other T helper cell subsets, and is required for the maintenance of their effector function in lymphoid organs (2–7). Activation of STAT3 is also important for Tfh differentiation as it upregulates BCL6 (8). STAT3-deficient mice have a diminished Tfh compartment and an increased Th1 response. The expression of BCL6 is antagonized by the transcriptional repressor, B lymphocyte-induced maturation protein 1 (BLIMP1) (4). Induction of BCL6 and the downregulation of BLIMP1 appear to be equally important in human Tfh differentiation (4, 8).

Follicular helper T cells express a unique set of effector molecules that are critical for their function. High expression of CXCR5 with downregulation of CCR7 has been shown to be important for migration of Tfh into the B cell follicle (9). Inducible co-stimulator (ICOS), programmed cell death protein 1 (PD-1), and CD40L are also expressed on Tfh cells and these molecules are required for activation of B cells (6, 10–14). Mice lacking CD40 or CD40L exhibit disrupted GC responses and impaired long-term memory (12). ICOS–ICOSL signaling is essential for a sustained T:B interaction; consequently, loss of either molecule impairs GC B cell survival and plasma cell differentiation (15). PD-1 deficiency does not affect the number of GC B cells and the early antibody response; however, engagement of PD-1 on Tfh cells and PD-L1/2 on GC B cells is critical for the development of long-lived plasma cells (13). Tfh cells produce high amounts of IL-21 and IL-4 which are required for proliferation of B cells and immunoglobulin class switching (6, 14). Blockade or deficiency of these effector molecules abrogates the generation of an effective humoral response.

## Induction Program of Tfh Cells

The immediate precursor of Tfh cells is not fully described. Initial studies identified the cytokines, IL-21 and IL-6, as key molecules in triggering Tfh cell differentiation. Engagement of TCR on naïve CD4^+^ T cells with peptide/MHC II on APCs together with IL-21 induces expression of CXCR5 and a low level of BCL6 expression *in vivo* (16, 17). Both IL-6 and IL-21 can induce *Bcl6* mRNA *in vitro* (5); both activate cells through the STAT3 pathway (8, 16). IL-6 and IL-21 can trigger the early Tfh differentiation program in CD4^+^ T cells, but an absolute requirement for IL-6 and IL-21 has been challenged. IL-6^−/−^, IL-21^−/−^, or IL-21R^−/−^ mice develop Tfh cells normally following immunization with protein antigen or viral infection (18, 19). Although the triggering signals for first induction of BCL6 and CXCR5 in Tfh cells are not fully understood, once T cells acquire a “CXCR5^lo^BCL6^lo^ Tfh signature (pre-Tfh),” some will migrate to the T cell–B cell border (9, 20). A second transcription factor, c-MAF, is induced concomitantly with BCL6 (21). C-MAF has also shown to induce CXCR5. BCL6 and c-MAF cooperatively induce ICOS, PD-1, and CXCR4, suggesting both molecules orchestrate a core transcriptional program in Tfh cells (22).

CXCR5^lo^BCL6^lo^ pre-Tfh cells interact with cognate B cells at the T–B zone to induce a high level of BCL6 and CXCR5. This allows stable localization of the cells in follicles and sustains mature Tfh cell differentiation (4, 23). Signaling from a homodimeric interaction of signaling lymphocytic activation molecule (SLAM)-associated protein (SAP) (SH2D1A) on B cells leads to induction of the SLAM family receptor, CD84, promoting stable T:B interactions (23, 24). In the absence of SAP, pre-Tfh cells develop normally, but fail to move into the GC and mature to GC-Tfh cells (24). This B cell-dependent Tfh differentiation can be bypassed by chronic immune activation. Mice lacking MHC II expression on B cells develop normal GC-Tfh cells following repeated immunization (25) or chronic viral infection (26). These observations suggest that while B cells maybe the major APC important in Tfh differentiation, B-independent Tfh maturation can occur when a high and sustained amount of antigen is present. An ICOS–ICOSL interaction between pre-Tfh and B cells is required for maintaining a high level of CXCR5 or BCL6 in Tfh cells. ICOS signaling activates the PI3K pathway and selective abrogation of ICOS–PI3K signaling dramatically reduces Tfh differentiation (27). ICOS–PI3K signaling keeps pre-Tfh cell motile at the T cell–B cell border to facilitate cognate T:B interactions (28). It also augments IL-4 and IL-21 transcription (27, 29). The importance of the PI3K pathway during Tfh differentiation is demonstrated in studies of mice with CD4-specific deletion of a microRNA miR 17-92. miR17-92 is induced at an early stage of Tfh cell differentiation and regulates PI3K signaling intensity through downregulation of phosphatase, PHLPP2. T cells with a deletion of miR17-92 show a severe reduction in Tfh differentiation (30).

## Negative Regulation of Tfh by Follicular Regulatory T (Tfr) Cells

The interaction between Tfh cells and B cells (GC B cells and plasma cells) needs to be precisely regulated to ensure proper immune activation and to limit excessive inflammation and autoimmunity. Tfr cells, a recently identified Treg subset, migrate to the GC and inhibit Tfh cells and GC B cells (31, 32). Differentiation of Tfr is mediated by recognition of antigens presented on DCs in lymphoid organs (31). Signals from the co-stimulatory molecules CD28 and ICOS are essential for Tfr differentiation as *Cd28*^−/−^ and *Icos*^−/−^ mice lack Tfr cells (32, 33). Engagement of CTLA-4 on Tfr cells with B7.1 and B7.2 on APCs is critical for their suppressive mechanism (34–36). In contrast, Tfr cells express high levels of PD-1 which mitigates the suppressive function of Tfr cells. *Pdcd1*^−/−^ Tfr cells suppress antibody production more potently *in vitro* and *in vivo* (33). Tfr cells express CXCR5 which guides them to the GC (32). Tfr, like Tfh cells, also express the canonical transcription factor, BCL6, although the level of BCL6 is lower than in Tfh cells. In addition to BCL6, Tfr cells express FOXP3 and BLIMP1, which are not expressed in Tfh cells (37).

The Tfh:Tfr ratio controls antibody responses. In the basal-state, Tfr cells constitute approximately 50% of all CD4^+^CXCR5^+^ T cells, resulting in a 1:1 ratio of Tfh:Tfr cells. Under stimulatory conditions including immunization or infection, Tfh cells expand resulting in a lower proportion of Tfr cells. A proper differentiation of Tfr is critical for immune tolerance as mice with Tfr deficiency (*Bcl6*^fl/fl^*Foxp3*-CRE) develop spontaneous autoimmune disease (38). A critical role for Tfr but not other Treg cells in antibody production was confirmed in an adoptive transfer study. Transfer of Tregs from Tfr-deficient Bcl6^−/−^ or wild-type mice together with CD4^+^ T cells into *Tcrb*^−/−^ mice resulted in an expansion of Tfh cells and higher antibody responses (37).

## Tfh in B Cell Activation and in GC and in the Extrafollicular Space

The primary function of Tfh cells is to help activation and differentiation of antigen-specific B cells in a protective immune response. This requires engagement of surface molecules (CD40L and ICOS) on Tfh cells with their ligands on B cells (CD40 and ICOSL, respectively) and secretion of cytokines (IL-21 and IL-4) from Tfh cells (6, 14). GCs are the primary site of B cell affinity maturation and class switching. Tfh cells regulate GC size, restrict low affinity B cell entry into the GC, and support and select high-affinity B cells during affinity maturation within the GC (4, 13, 29, 39). The quality and quantity of help provided by Tfh cells regulates B cell clonal expansion. As restriction of T cell help to high-affinity B cells is required for affinity maturation in GCs (40), proper regulation of Tfh determines the outcome of the GC response.

Signals from Tfh cell to B cells are required for both the generation and the maintenance of GCs. To initiate GC B cell differentiation, Tfh cells induce expression of BCL6 (master transcription factor for GC B cells) in activated B cells. The precise mechanism how the initial BCL6 expression occurs is complex and not clearly understood; however, signals from the IL-21R are an important factor for BCL6 expression in B cells (18, 41). Tfh cells also provide proliferation and survival signals to GC B cells *via* multiple pathways, including CD40L, PD-1, IL-21, and IL-4 (41–44). The CD40–CD40L interaction is important in survival of GC B cells partly because it also helps to induce BCL6 (45). Combinatorial signals by CD40L and IL-21 or CD40L and IL-4 maintain GC B cell proliferation. Although PD-1 is known to provide a potent inhibitory signal to T cells (46), deficiency in PD-1 or PD-L1/2 reduces B cell differentiation (13). Formation of GCs is normal in the absence of PD-1 or PD-L1/2, but maintenance of GCs is severely affected due to an increase in apoptosis of GC B cells. The interaction between ICOS on Tfh cells and ICOSL on B cells is important for both B cells and Tfh cells (10, 11). The ICOS–ICOSL signaling is essential for the sustained T:B interaction, consequently influencing affinity maturation of GC B cells, survival, and plasma cell differentiation (15). Cognate interaction between Tfh and GC B cells is a key mechanism for selection of high-affinity GC B cells and for memory B cells or plasma cells (47, 48). Tfh cells regulate plasmablast emergence out of GC during the early stages of GC reaction. IL-21 produced from Tfh cells and TNFSF13 (APRIL) produced form podoplanin^+^ CD157^hi^ fibroblastic reticular cells are the two main factors for this process (49). IL-21 is highly expressed by Tfh cells and is the most potent cytokine for driving plasma cell differentiation (2, 50). Both IL-21 and IL-4 are class switch factors for IgG1 (51, 52).

Follicular helper T cells play a key role in B cell differentiation into antibody-producing cells outside GCs as well. Recent studies demonstrate that there are different subsets of Tfh cells in humans and mice. BCL6^+^ Tfh cells are required for B cell priming for extrafollicular antibody production in a T-dependent immune response (53). In contrast to the conventional Tfh cells, they express PD-1 but not CXCR5 and appear before GC formation at the T cell–B cell border. IL-21 produced from these PD-1^+^ Tfh cells support B cell activation and differentiation to antibody-producing cells.

## Repertoire of Tfh

How the repertoire of Tfh cells is determined is not well understood, and an altered repertoire of Tfh cells can contribute to the development of autoimmune diseases. Our recent study demonstrated that the repertoire of Tfh is different in lupus-prone mice compared to healthy control mice, and the alteration of Tfh repertoire is closely associated with lupus development (54). This study suggests that not only the number of Tfh cells, but also the antigenic specificity of Tfh cells is likely essential to immune tolerance.

Sets of antigenic peptides presented by APCs, including thymic epithelial cells, dendritic cells (DCs), and B cells are likely to influence the repertoire of T cells emerging from the thymus. In the periphery, affinity and duration of interaction between TCR and peptide/MHC II influences DCs to induce the activation of T cells and modulate the repertoire of T cells (25, 55). Cathepsin S (CTSS) is a major endoprotease cleaving the invariant chain from MHC II molecules and also cleaving exogenous antigens (54). Increased activity of CTSS in DCs is involved in regulation of the Tfh repertoire (54). In one study, the antigenic specificity of Tfh cells was investigated during a polyclonal B cell response in mice (56). Polyreactive Tfh cells are generated and individual antigen-specific Tfh cells show distinct cytokine profiles. A study of human Tfh shows that CXCR5^+^ circulating memory-like Tfh cells reactive with influenza protein preferentially recognize peptide epitopes from hemagglutinin, while CXCR5^−^ non-Tfh preferentially recognize nucleoprotein (57). This study suggests that different effector T cell subsets may activate distinct B cells. Together, these observations, while limited, support an important role for the TCR repertoire of Tfh cells in autoreactive B cell selection and/or activation.

## Human Tfh

Human Tfh cells are characterized in tonsil by high expression of CXCR5 and ICOS (58). These CXCR5^hi^ICOS^hi^ cells reside in follicles and induce B cells to become antibody-secreting plasma cells, which is a hallmark of *bona fide* Tfh cells. In healthy individuals, tonsillar CD4^+^ T cells contain distinct populations according to the expression of CXCR5 and ICOS, including CXCR5^hi^ICOS^hi^ (GC-Tfh), CXCR5^lo^ICOS^hi^, CXCR5^lo^ICOS^lo^, and CXCR5^−^ICOS^−^. These subpopulations of Tfh cells display differential activation of B cells. CXCR5^hi^ICOS^hi^ Tfh cells effectively activate and induce antibody production of GC-B cells (IgD^−^CD38^+^CD19^+^) and memory B cells (IgD^−^CD38^−^CD19^+^) but not naïve B cells (IgD^+^CD38^−^CD19^+^) (58, 59). CXCR5^lo^ICOS^lo^ Tfh cells show robust proliferation and differentiation of naïve B cells and memory B cells but not GC B cells (60). Neither CXCR5^−^ICOS^−^ Tfh nor CXCl5^lo^ICOS^hi^ Tfh cells show B cell activation activity. Each subpopulation of Tfh cells also produces a distinct pattern of cytokines. Upon co-culture with B cells or stimulated by anti-CD3/28, CXCR5^hi^ICOS^hi^ GC-Tfh cells secrete high level of IL-21 and CXCL13 but low level of IL-10 and IL-4, CXCR5^lo^ICOS^lo^ Tfh cells secrete high levels of IL-21 and IL-10 with low level of IL-4, and CXCR5^lo^ICOS^hi^ Tfh cells secrete high level of IL-17A instead of IL-21 and IL-10. These studies suggest each Tfh population possesses a unique B cell activation capacity.

**Figure 1:**
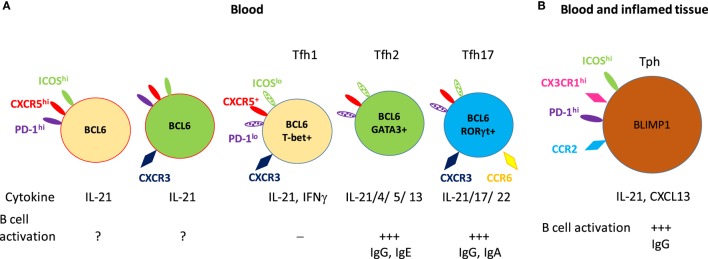
Subsets of circulating memory follicular helper T (Tfh) cells and their B cell activation. **(A)** The circulating CXCR5^+^ memory Tfh subsets. The minor CXCR5^+^ CD4^+^ T cells (1–5% of CXCR5^+^CD4^+^CD4^+^ T cells) express high levels of inducible co-stimulator (ICOS) and programmed cell death protein 1 (PD-1) and consist of CXCR3-positive or CXCR3-negative populations. The majority of CXCR5^+^ CD4^+^ T cells are low to negative in expression of ICOS and PD-1. These cells are subgrouped into Tfh1, Tfh2, and Tfh17 based on their chemokine receptor expression. These five subsets of Tfh cells express B cell lymphoma 6 as a master transcriptional regulator, and also express subset-specific transcription factors, effector cytokines, and activate naïve B cells to antibody-secreting cells. **(B)** CXCR5^−^ CD4^+^ T cells [peripheral helper T (Tph)] which express high level of PD-1 exhibit strong B cell activation. These Tph cells developed under inflamed conditions and are found in both inflamed tissue and blood. Solid color indicates high level of expression and patterned color indicates low level of expression.

B cell lymphoma 6-expressing extrafollicular Tfh cells have been identified in tissues and in blood (60, 61). Tfh cells identified in blood are different from tonsillar Tfh cells phenotypically and functionally. Circulating Tfh cells do not have same patterns of surface markers (CXCR5, ICOS, and PD-1) as tonsillar Tfh cells. Recent studies suggest that blood CXCR5^+^ CD4^+^ T cells represent a memory compartment of Tfh lineage cells. Extensive analysis of these cells revealed functionally and phenotypically distinct subsets, identified by expression of ICOS, PD-1, CCR7, CXCR3, and CCR6. While the expression of BCL6 and ICOS is high in tonsillar GC-Tfh cells, both ICOS and BCL6 protein expression is low in blood Tfh cells (61, 62). Less than 1% of blood CXCR5^+^ Tfh cells are ICOS^+^ and PD-1^hi^ in healthy individuals (63, 64). The majority of CXCR5^+^ Tfh cells does not express ICOS and can be subsetted based on CXCR3, CCR6, and CCR7 expression, and, as mentioned above, each subgroup activates distinctive B cell subsets and expresses different gene expression profiles (Figure 1). The CXCR3^+^ CCR6^−^ subset (Tfh1) expresses the transcription factor T-bet and produces IFNγ; the CXCR3^−^ CCR6^−^ subset (Tfh2)expresses GATA3 and produces IL-4, IL-5, and IL-13; and the CXCR3^−^ CCR6^+^ subset (Tfh17) expresses RORγt and produces IL-17A and IL-22 (61). All subsets of Tfh cells produce IL-21 but not all induce B cells to secrete immunoglobulin; each subset has a distinct capacity for B cell activation. Naïve B cells can be stimulated by Tfh2 and Tfh17 but not CXCR3^+^ Tfh1 cells (61). Tfh2 cells promote IgG and IgE, while Tfh17 cells efficient inducers of IgG and IgA production. These studies suggest different Tfh subsets regulate Ig class switching these subsets appear to exist in humans but not in mice.

The mechanism of human Tfh cell differentiation is actively being investigated. Human Tfh cells are differentiated from naïve CD4^+^ T cells, however, the critical signals and effector molecules are not fully understood. The plasticity among CD4^+^ helper T cell subsets is also poorly understood. Recent *in vitro* studies identified some molecules which influence human Tfh differentiation. Initial studies demonstrated that DCs can induce differentiation of Tfh cells from naïve CD4^+^ T cells from human peripheral blood mononuclear cell (PBMCs) or from cord blood. Among the cytokines which are produced from activated DCs, IL-12 is the most efficient cytokine to induce Tfh-related molecules (CXCR5, ICOS, and IL-21) and Tfh-related transcription factors (BCL6, BATF, and cMAF) (65). Another study found IL-12 most efficiently induced Tfh cells to produce IL-21 to activate B cells to become antibody-secreting cells (66). Surprisingly, these studies found that the Tfh-inducing cytokines in mouse, IL-6 or IL-21, are less efficient in the human system. IL-12 mediated human Tfh cells contain classical Th1 phenotypes, displaying a mixed population of IL-21^+^IFNγ^−^ Tfh and IL-21^+^IFNγ^+^ Tfh cells. The importance of IL-12 in Tfh cell differentiation is also supported by the studies of patients with impaired IL-12 signaling. Patients with deficiency in IL-12Rβ1, TYK2, STAT3, but not STAT1 exhibit compromised IL-12-induced expression of IL-21 by CD4^+^ T cells (67). Although IL-12/STAT3 axis is critical for IL-21 and BCL6 expression, it is a dispensable for ICOS expression. Defects in generating Tfh cells from STAT3 mutant CD4^+^ T cells could contribute to the impaired T-dependent humoral immune responses observed in patients with STAT3 mutations (68).

Another cytokine, TGFβ, was shown to have a unique function in human Tfh cell differentiation (69). TGFβ and IL-12 together specifically down-regulate the level of BLIMP1 in Tfh cells. This regulatory mechanism is not shared by mouse Tfh cells. TGFβ also induces CXCL13 expression in human naïve CD4^+^ T cells (70), and, therefore, may contribute to the accumulation of T and B cell aggregates (ectopic GCs) in inflammatory tissues (71).

Activin A is an inducible molecule that is broadly expressed in immune cells. Signals *via* CD40 and toll-like receptors induce DCs to upregulate Activin A expression (72); Activin A induces human naïve CD4^+^ T cells to produce CXCL13 and differentiate into Tfh cells *in vitro* (73). Therefore, Activin A may be also involved in Tfh differentiation especially under inflammatory conditions. A recent study on systemic lupus erythematosus (SLE) suggests the involvement of OX40L^+^ monocytes and DCs in Tfh production. The frequency of OX40L^+^ monocytes correlated with disease activity and the frequency of ICOS^+^ PD-1^+^ Tfh cells in blood (74). TNFSF4, the gene encoding OX40L, has been determined by GWAS to have risk alleles in SLE, rheumatoid arthritis (RA), and multiple sclerosis (75), further suggesting an involvement of OX40 and OX40L interaction mediated Tfh pathway in disease conditions. The molecular mechanisms by which naïve human CD4^+^ T cells differentiate to Tfh cells or maintain Tfh characteristics remain largely unknown.

## Relevance to Autoimmunity

Helper T cells are required for a protective immune response and for the development of autoantibodies (76). Development of autoantibodies is abrogated by genetic deletion of MHC II in CD4^+^ T cells in the B6. *lpr* lupus mouse model (77). Helper T cell activity, especially Tfh cell activity, directly correlates with GC formation (78–80). Abnormalities in Tfh cells (Tfh cell accumulation or functional alteration of Tfh cells) have been observed in various models of autoimmune diseases. In *sanroque* mice, the number of Tfh cells is increased with the hyperactive ICOS signaling and excessive production of IL-21, leading to the development of spontaneous GC formation and lupus-like autoimmune phenotypes (3, 81, 82). Roquin has been shown to post-transcriptionally regulate the expression of ICOS and OX40, which are highly expressed by Tfh cells (58, 81, 83). CD4+ T cells lacking Roquin overexpress ICOS and OX40, promoting Tfh cell differentiation. Blocking ICOS/ICOS-L or CD40/CD40L interactions ameliorates disease progression in autoimmune mouse models (84, 85). High serum levels of IL-21 were also detected in BXSB-Yaa mice (2), and blockade of the IL-21/IL-21R pathway slows the progression of lupus by decreasing lymphocyte activation and circulating IgG1 levels in BXSB-Yaa mice (86). These data demonstrate IL-21 as a key molecule for B cell differentiation to plasma cells. In this model, both follicular and extrafollicular T cells are important producers of IL-21. Another SLE model, MRL/lpr mice, has increased extrafollicular ICOS^hi^PSGL1^lo^ CD4^+^ T cells in secondary lymphoid organs. These cells are the primary source of CD40L and IL-21 supporting extrafollicular IgG plasmablasts (10). CD40L expressed by Tfh cells contributes to activation of B cells in autoimmune animal models. Antigen-specific extra Tfh cells which share phenotypic characteristics with Tfh also mediate IgG secretion through IL-21 in a CD40L-dependent manner (10). Autoreactive Tfh cells have been observed in K/BxN mice, too. In this mice, self-reactive CD4^+^ T cells escape clonal deletion in the thymus and appear in periphery. The primed self-reactive CD4^+^ T cells help B cells to produce pathologic antibodies (87). Tfh cells have been shown to contribute to the pathogenesis of lupus through ICOS–B7RP-1 pathway in NZB/NZW F1 mice (88). Abnormalities in Tfh cells are also observed in mice with genetic manipulations in non-T cells. Mice with BLIMP1-deficient DCs spontaneously develop lupus-like phenotypes in a gender-specific manner, and these mice have increased Tfh cells and GC B cells (89). This study also shows that an increased proinflammatory cytokine, IL-6 from the BLIMP1-deficient DCs facilitates Tfh differentiation as haplosufficiency for IL-6, prevents lupus-related phenotypes including Tfh cells.

There are multiple lines of evidences showing that aberrant Tfh-cell and GC responses are associated with human SLE. The majority of IgG+ autoantibodies in patients are somatically mutated, an observation consistent with an involvement of GCs, the site of action of Tfh (90, 91). In lupus nephritis lesions, Tfh-like cells expressing ICOS, PD-1, BCL-6, and IL-21 were observed, forming ectopic GCs (92). Furthermore, an increased population of circulating CXCR5^+^ICOS^+^PD-1^+^ Tfh cells was identified in a subset of SLE patients; this increase correlated with disease activity but not necessarily with the titer of anti-DNA antibodies (93, 94). ICOS^+^PD-1^+^ Tfh cells secrete high levels of IL-21, and a strong correlation between the expression of ICOS or PD-1 and plasmablast number has been observed. The differential expression of ICOS, PD-1, and CCR7 further defines subpopulations within the subsets (recently activated: ICOS^+^PD-1^+^CCR7^lo^ and quiescent: ICOS^−^PD-1^−^CCR7^hi^) (62, 63). Other studies employing a combinatorial analysis of chemokine receptors (CXCR3 and CCR6) have defined three major subsets (Tfh1, Tfh2, and Tfh17) (61, 69). These analyses identified a relative dominance of Tfh2 and/or Tfh17 subsets over Tfh1 in systemic diseases, including SLE (93, 95), IgG4-related disease (96), and organ-specific diseases such as [Sjogren’s syndrome (97), RA (98, 99), and autoimmune thyroid disease (100)], and the neurological diseases [myasthenia gravis (101), multiple sclerosis (102), and neuromyelitis optica (103)]. The alterations in Tfh subsets often positively correlated with disease activity and/or serum autoantibody titer, and with the frequency of circulating plasmablasts. These observations support the association of an expanded Tfh response with the pathogenesis of human autoimmune diseases; however, how the increased Tfh response leads to activation of autoreactive B cells is not elucidated in humans yet.

A recent study identified a new PD-1^hi^ helper T cell subset in peripheral tissue [peripheral helper T (Tph) cells] from patients with RA (104). Tph cells are expanded in inflamed joint and blood from RA patients and display unusual biological features. They are programmed to infiltrate part of the inflamed body (CCR2^+^), and stimulate B cells to produce antibodies (IL-21 and CXCL13) *in situ*. In contrast to CXCR5^+^ Tfh cells, Tph cells exhibit a unique profile of molecules; CXCR5^−^ but BLIMP1^hi^ (high ratio of BLIMP1:BCL6) and PD-1^hi^ (104). This finding expands the spectrum of T cells that are associated with inflammatory diseases; it will be interesting to investigate whether PD-1^hi^ Tph cells are expanded in other autoimmune and inflammatory diseases.

## Conclusion

Compelling data now demonstrate the key role of the Tfh in B cell responses, both protective and pathogenic. Much still remains to be learned. For example, we do not know whether the subsets we have identified represent activation or differentiation states, and whether there is phenotypic and functional plasticity among any of these subsets. We do not know how Tph relate to Tfh and whether and how they contribute to tissue inflammation. We need to learn whether uncontrolled antibody responses relate to an expanded Tfh number or an altered Tfh repertoire and we need to learn if Tfh selectively respond to particular antigens. The field is rapidly growing and answers to these, and answers to these questions, and more, are likely to be forthcoming and to suggest strategies for treatment of both autoimmune diseases and immunodeficiency states.

## Author Contributions

BD organizes and supervises the entire manuscript. SK contributes for human section. KL contributes for mouse section.

## Conflict of Interest Statement

The authors declare that the research was conducted in the absence of any commercial or financial relationships that could be construed as a potential conflict of interest.
